# Hyperammonemic Encephalopathy Caused by Obstructive Urinary Tract Infection Due to Urease-Positive Corynebacterium riegelii in an Elderly Woman: A Case Report

**DOI:** 10.7759/cureus.97833

**Published:** 2025-11-26

**Authors:** Tomoyuki Araya, Toshiyuki Kita, Takayuki Higashi, Ryo Hara, Hazuki Takato

**Affiliations:** 1 Respiratory Medicine, National Hospital Organization (NHO) Kanazawa Medical Center, Kanazawa, JPN

**Keywords:** corynebacterium riegelii, hyperammonemia-encephalopathy, multidisciplinary approach, urease-producing bacteria, urinary tract infection

## Abstract

Hyperammonemia typically results from hepatic dysfunction, but obstructive urinary tract infection (UTI) caused by urease-producing bacteria can rarely induce severe hyperammonemia and encephalopathy. We describe a 76-year-old woman with chronic voiding dysfunction who presented with fever and coma, with a markedly elevated serum ammonia level of 380 μg/dL despite normal liver function. Urinalysis revealed markedly alkaline urine and pyuria. Head computed tomography (CT) and magnetic resonance imaging showed no acute abnormalities. Abdominal CT demonstrated marked bladder distention and no findings suggestive of chronic liver disease. Bladder decompression and piperacillin/tazobactam therapy led to rapid improvement, with ammonia falling to 70 μg/dL and full recovery of consciousness within 48 hours. Urine culture later yielded *Corynebacterium* species, prompting the addition of vancomycin; the organism was subsequently identified as urease-positive *Corynebacterium* *riegelii*. Blood cultures remained negative, and a later plasma amino acid analysis showed no evidence of urea-cycle disorders. The patient had no recurrence of hyperammonemia or UTI during the four-year follow-up. Obstructive UTI due to urease-producing bacteria should be recognized as a reversible cause of acute encephalopathy in elderly patients with urinary retention.

## Introduction

Non-hepatic hyperammonemia secondary to urinary tract infection (UTI) is an important yet often underrecognized cause of altered consciousness [1-3]. Obstructive UTI caused by urease-producing organisms can elevate intravesical pressure, facilitating ammonia back-diffusion through the vesical venous plexus into the systemic circulation, thereby bypassing hepatic detoxification and resulting in significant hyperammonemia and encephalopathy [2-4].

Representative urease-producing uropathogens include *Proteus mirabilis, Providencia stuartii, Morganella morganii, Staphylococcus saprophyticus*, and *Corynebacterium urealyticum*, all of which have been well characterized clinically [5,6]. Among *Corynebacterium* species, *Corynebacterium riegelii* has emerged as a urease-producing pathogen of clinical relevance [7]. It is most frequently isolated from female patients with UTIs and is notable for its strong urease activity, which may contribute to alkaline urine, urinary calculi, and, in some cases, obstructive complications or hyperammonemia. Although usually associated with localized urinary infections, *Corynebacterium riegelii* can occasionally cause severe or even life-threatening infections, including urosepsis [8]. Rather than its morphological traits, the clinical relevance of Corynebacterium riegelii lies in the fact that it is often overlooked or misidentified in routine laboratories due to its resemblance to other coryneform bacteria. Reliable identification, therefore, frequently requires advanced methods such as matrix-assisted laser desorption/ionization time-of-flight (MALDI-TOF) mass spectrometry [7-9].

Management of obstructive UTI-related hyperammonemia requires prompt relief of urinary tract obstruction in combination with appropriate antimicrobial therapy. Decompression with catheterization or drainage, with surgical intervention when necessary, effectively reduces intravesical pressure and limits further ammonia production and absorption [2,3]. When recognized early, hyperammonemia generally improves rapidly once obstruction is relieved and infection controlled; however, prognosis may be adversely affected when serious complications such as aspiration pneumonia occur, even with adequate treatment [2,3].

We report an elderly woman who developed severe hyperammonemia with coma secondary to urinary retention, caused by the urease-producing uropathogen *Corynebacterium riegelii*, and was successfully treated with prompt bladder decompression and appropriate antimicrobial therapy.

## Case presentation

A 76-year-old woman residing in a nursing facility had a history of femoral neck fracture one year earlier, after which she developed persistent impairment in activities of daily living and chronic voiding dysfunction characterized by functional obstructive lower urinary tract symptoms due to reduced mobility, without evidence of anatomical obstruction. She had no history of liver disease, took no regular medications, and had no exposure to drugs known to elevate ammonia levels. She did not smoke or consume alcohol, and her family history was unremarkable.

She was found febrile and unresponsive at her facility and was transported to our hospital. Although she was febrile, there was no history of chills or rigors. On arrival, her vital signs were as follows: temperature 37.6°C, blood pressure 138/86 mmHg, pulse 105/min, respiratory rate 22/min, and SpO₂ 98% on room air. She was comatose, corresponding to a Glasgow Coma Scale (GCS) score of 6 (E1V1M4). Physical examination revealed marked lower abdominal distension without jaundice, meningeal signs, or cardiopulmonary abnormalities. A focused female pelvic examination revealed no vulvar abnormalities, no external urethral meatal lesions, and no vaginal discharge. There was no evidence of pelvic organ prolapse or gynecologic conditions that could contribute to urinary retention. Neurological examination demonstrated no focal deficits; ocular movements were intact, deep tendon reflexes were normal, and no pathological reflexes were present.

Initial laboratory evaluation demonstrated leukocytosis (white blood cell count 14,100/µL) and markedly elevated C-reactive protein (14.54 mg/dL), along with a profoundly elevated serum ammonia level of 380 μg/dL (reference 12-66 μg/dL) despite preserved hepatic function. Urinalysis revealed alkaline urine (pH 8.5) with proteinuria (1+), hematuria (3+), and pyuria (Table 1).

**Table 1:** Laboratory findings on the day of admission Laboratory data obtained on the day of admission showed marked leukocytosis, elevated C-reactive protein and D-dimer levels, and severe hyperammonemia, whereas liver enzymes and bilirubin were within or close to the normal ranges, indicating the absence of overt hepatic dysfunction. Urinalysis demonstrated alkaline urine with proteinuria, hematuria, and pyuria, findings suggestive of a urinary tract infection. Values are presented alongside their corresponding reference ranges. WBC: white blood cell; Neu: neutrophil; Lym: lymphocyte; Mon: monocyte; Eos: eosinophil; Bas: basophil; RBC: red blood cell; Hb: hemoglobin; Hct: hematocrit; PLT: platelet; CRP: C-reactive protein; T-Bil: total bilirubin; D-Bil: direct bilirubin; I-Bil: indirect bilirubin; TP: total protein; Alb: albumin; ALP: alkaline phosphatase; AST: aspartate aminotransferase; ALT: alanine aminotransferase; γ-GTP: gamma-glutamyl transpeptidase; LDH: lactate dehydrogenase; Na: sodium; K: potassium; Cl: chloride; BUN: blood urea nitrogen; Cr: creatinine; eGFR: estimated glomerular filtration rate; UA: uric acid; CK: creatine kinase; Amy: amylase; P-Amy: pancreatic amylase; BNP: brain natriuretic peptide; PT-INR: prothrombin time-international normalized ratio; CEA: carcinoembryonic antigen; AFP: alpha-fetoprotein; HPF: high-power field; SG: specific gravity; HBs Ag: hepatitis B surface antigen; HCV Ab: hepatitis C virus antibody.

Parameter (Unit)	Value	Reference Range
WBC (/µL)	14,100	4500-9000
Neu (%)	93.0	38-74
Lym (%)	3.0	16.5-49.5
Mon (%)	4.0	5-10
Eos (%)	0	0-10
Bas (%)	0	0-2
RBC (×10⁴/µL)	392	382-500
Hb (g/dL)	14.0	11.7-14.6
Hct (%)	38.8	34.3-44.2
PLT (×10⁴/µL)	29.4	15-35
CRP (mg/dL)	14.54	0-0.4
Procalcitonin (µg/mL)	0.11	<0.5
T-Bil (mg/dL)	1.6	0.3-1.2
D-Bil (mg/dL)	0.1	0-0.2
I-Bil (mg/dL)	1.4	0.2-1.1
TP (g/dL)	6.3	6.7-8.3
Alb (g/dL)	4.4	4.0-5.0
ALP (U/L)	99	38-113
AST (U/L)	19	13-33
ALT (U/L)	18	6-27
γ-GTP (IU/L)	22	10-47
LDH (U/L)	207	119-229
Na (mEq/L)	136	135-149
K (mEq/L)	4.4	3.5-4.9
Cl (mEq/L)	105	96-108
BUN (mg/dL)	23.0	8-22
Cr (mg/dL)	0.42	0.5-0.8
eGFR (mL/min)	106.9	60-100
UA (mg/dL)	2.8	2.3-7.0
CK (U/L)	98	45-163
Amy (U/L)	98	35-140
P-Amy (U/L)	56	15-65
BNP (pg/mL)	19.9	<18.4
Lactic acid (mmol/L)	1.8	1.0-1.5
HbA1c (%)	5.4	<6.4
PT-INR	0.88	0.8-1.2
D-dimer (µg/mL)	4.2	0-1
Ammonia (µg/dL)	380	12-66
CEA (ng/mL)	4.6	<3.5
AFP (ng/mL)	1	<10
CA19-9 (U/mL)	22	<35
IgG (mg/dL)	753	870-1700
IgA (mg/dL)	68	110-410
IgM (mg/dL)	39	35-220
HBs Ag	Negative	Negative
HCV Ab	Negative	Negative
Urine pH	8.5	4.5-8.0
Urine SG	1.015	1.002-1.030
Urine protein	1+	Negative
Urine glucose	Negative	Negative
Urine occult blood	3+	Negative
Nitrite	Negative	Negative
Urine WBC	3+	Negative
Sediment WBC (/HPF)	>100	0-4
Sediment RBC (/HPF)	50-99	0-4

**Figure 1:**
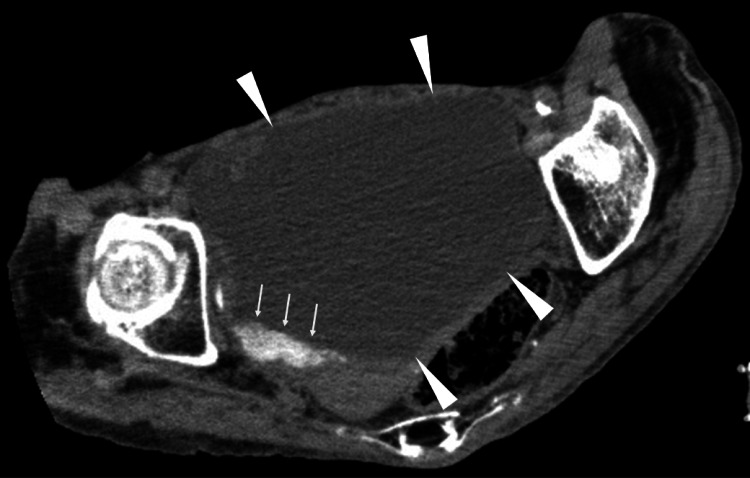
Markedly distended bladder with urinary stasis Abdominal computed tomography demonstrates a markedly distended bladder with significant urinary stasis (arrowheads) and abundant intravesical sediment (arrows). These findings are consistent with chronic voiding dysfunction and obstructive urinary retention.

Abdominal computed tomography (CT) showed a markedly distended bladder with urinary stasis and intravesical sediment, and the kidneys and upper urinary tracts showed no hydronephrosis or other abnormalities (Figure 1). The abdominal CT also showed no findings suggestive of chronic liver disease. Head CT revealed no intracranial hemorrhage, and magnetic resonance imaging of the brain demonstrated no acute diffusion-restricted lesions, showing only chronic ischemic white-matter changes.

The differential diagnosis included hepatic encephalopathy, urea-cycle disorders, and infection-related causes, all considered as potential explanations for the severe hyperammonemia and coma. Despite normal hepatic function, hepatic encephalopathy was initially suspected. Although neither liver biochemistry nor abdominal imaging supported overt hepatic disease, gastroenterology and endocrinology specialists were consulted, and hepatic and metabolic etiologies could not be definitively excluded until the results of comprehensive liver function testing and screening for amino acid metabolic disorders became available. During this evaluation period, the patient was treated with intravenous amino acid therapy enriched with branched-chain amino acids together with the non-absorbable antibiotic rifaximin administered via a nasogastric tube, while concurrently undergoing bladder decompression with immediate placement of an indwelling urethral catheter and receiving empirical piperacillin/tazobactam for the suspected urinary tract infection. A 14 Fr urethral catheter was inserted without difficulty, and 700 mL of urine was drained immediately, indicating marked acute-on-chronic urinary retention. The urine was strongly turbid with abundant floating sediment and a pronounced malodor, consistent with infected stagnant urine. No obvious post-obstructive diuresis was observed, and following catheterization, the patient maintained a daily urine output ranging from 1000 to 2000 mL during hospitalization.

Her serum ammonia level declined rapidly from 380 to 70 μg/dL within 48 hours, with a corresponding improvement in consciousness to a normal level (GCS 15). Blood cultures obtained at admission were negative. Urine culture grew *Corynebacterium* species on hospital day 4, prompting the addition of vancomycin, and she defervesced the same day. Ammonia levels normalized to 22 μg/dL by day 7. By this time, the results of the outsourced immunological and liver-related tests had become available and excluded underlying hepatic disease (Table 2). In addition to the improvement in hyperammonemia, intravenous branched-chain amino acid-enriched amino acid therapy and rifaximin were discontinued. The organism was subsequently identified as urease-producing Corynebacterium riegelii on day 8, and all antibiotics were discontinued on day 9. Plasma amino acid analysis did not indicate a urea-cycle disorder. The patient experienced no recurrence of hyperammonemia, completed rehabilitation, and was discharged on day 42.

**Table 2:** Immunological tests drawn on admission (day 0) and reported on day 7 These blood test results, including autoimmune and immunological assays, were obtained on the day of admission (Day 0) but were reported on Day 7 due to external laboratory processing time. The findings showed no evidence of underlying hepatic disease, supporting the exclusion of liver-related causes of hyperammonemia. Values are presented along with their corresponding reference ranges. ANA, antinuclear antibody; ASMA, anti-smooth muscle antibody.

Parameter (Unit)	Value	Reference Range
ANA (titer)	<40	<40
Anti-mitochondrial antibody	Negative	Negative
Anti-mitochondrial M2 antibody (U/mL)	1.5	0–7.0
ASMA	Negative	Negative

After discussion with the urology team, long-term catheterization was selected because removal of the indwelling urethral catheter was judged to carry a substantial risk of recurrent urinary retention and subsequent hyperammonemic episodes, potentially leading to a fatal course. During the acute phase, a formal urodynamic evaluation was not performed because the patient presented in a comatose state with severe hyperammonemia, and priority was placed on life-saving management. Given her advanced age and severe impairment in activities of daily living, clean intermittent catheterization was considered impractical, and the urology team recommended long-term indwelling catheterization. Following thorough shared decision-making with the patient, a permanent catheter placement was chosen. During hospitalization, standard catheter-associated infection prevention measures were strictly implemented, including maintenance of a closed drainage system, routine perineal hygiene performed by nursing staff, and scheduled drainage bag replacement according to institutional protocol. At the time of discharge, the nursing facility staff were instructed to continue these preventive strategies to minimize the risk of catheter-associated urinary tract infection. Over the subsequent four years, she has remained free of recurrent urinary tract infections or any readmissions for altered consciousness.

## Discussion

This case highlights a reversible, non-hepatic cause of severe hyperammonemia with coma: obstructive urinary tract infection due to a urease-producing organism. Although uncommon, timely recognition is clinically crucial, yet the diagnosis can be challenging in real-world practice, particularly when multiple competing etiologies of hyperammonemia must be evaluated simultaneously. In obstructive UTI caused by urease-producing organisms, intravesical ammonia production markedly increases, and elevated bladder pressure facilitates ammonia back-diffusion through the vesical venous plexus into the systemic circulation, effectively bypassing hepatic detoxification and resulting in severe hyperammonemia [2-4].

*Corynebacterium riegelii* possesses several pathogenic characteristics that are clinically relevant in urinary tract infections. Beyond its strong urease activity, which promotes urinary alkalinization and predisposes to crystal and calculus formation, this organism has been reported to cause symptomatic infections in both immunocompetent and immunocompromised hosts [7,8]. It is most frequently isolated from older women with voiding dysfunction, and cases ranging from localized cystitis to severe urosepsis have been documented. Because routine biochemical systems often misidentify coryneform bacteria, the pathogenic potential of *Corynebacterium riegelii* may be underrecognized, particularly in patients with chronic urinary stasis in whom the organism can proliferate and generate substantial intravesical ammonia.

These pathogenic attributes underscore the importance of considering *Corynebacterium riegelii* in the differential diagnosis of obstructive UTI-related hyperammonemia. Hyperammonemic encephalopathy attributable to *Corynebacterium riegelii* is exceptionally rare; only isolated case reports exist, including one described by Yada et al. [10]. Their patient experienced acute urinary retention with rapid improvement following decompression, without chronic voiding dysfunction, diagnostic complexity, or extended follow-up. In contrast, the present case involved a longstanding voiding impairment, a more protracted and multifaceted diagnostic pathway, and a gradual recovery trajectory. Furthermore, while previous reports have not involved multidisciplinary evaluation, coordinated assessment by gastroenterology, endocrinology, and urology specialists was essential in this case to exclude hepatic and metabolic causes, confirm the infective etiology, and determine a safe long-term management strategy.

Accurate identification of *Corynebacterium riegelii* can be difficult with conventional biochemical methods. MALDI-TOF MS has been reported as a reliable tool for species-level identification, as demonstrated by Pichon et al. [9], and can facilitate appropriate antimicrobial selection. Clinicians should remain alert to key diagnostic clues that may point toward obstructive UTI-related hyperammonemia: unexplained severe hyperammonemia with altered consciousness, chronic voiding dysfunction, marked bladder distention on CT, and alkaline urine with pyuria [11,12]. When these features coexist, and alternative hepatic or metabolic causes cannot account for the degree of hyperammonemia, obstructive UTI due to a urease-producing organism should be actively considered.

The rapid biochemical and neurological improvement following bladder decompression and antimicrobial therapy underscores the importance of early recognition in achieving an excellent prognosis [2]. Nevertheless, empirical antibiotic therapy must be initiated judiciously, ensuring coverage of likely urease-producing uropathogens while permitting timely refinement according to culture and susceptibility results [13]. Early measurement of serum ammonia, prompt imaging to evaluate bladder distention, and immediate decompression remain essential components of management in elderly patients presenting with urinary retention and acute encephalopathy.

## Conclusions

This case demonstrates that obstructive urinary tract infection caused by a urease-producing organism can lead to severe but reversible hyperammonemic encephalopathy, even in the absence of liver disease. Recognition of key diagnostic clues-unexplained marked hyperammonemia, chronic voiding dysfunction, significant bladder distention on imaging, and alkaline urine with pyuria-is essential for timely diagnosis. Early bladder decompression combined with appropriate antimicrobial therapy can result in rapid neurological recovery and an excellent prognosis. Given the diagnostic complexity associated with this condition, multidisciplinary evaluation plays a critical role in excluding alternative etiologies, guiding management decisions, and preventing recurrence.
